# Ni-hemin metal–organic framework with highly efficient peroxidase catalytic activity: toward colorimetric cancer cell detection and targeted therapeutics

**DOI:** 10.1186/s12951-018-0421-7

**Published:** 2018-11-20

**Authors:** Negar Alizadeh, Abdollah Salimi, Rahman Hallaj, Fardin Fathi, Farzad Soleimani

**Affiliations:** 1 Department of Chemistry, University of Kurdistan, 66177-15175 Sanandaj, Iran; 2 Research Center for Nanotechnology, University of Kurdistan, 66177-15175 Sanandaj, Iran; 3 Cellular and Molecular Reserch Center, Kurdistan University of Medical Sciences, 66177-13446 Sananandaj, Iran

**Keywords:** Ni-hemin MOF, Peroxidase activity, TMB, H_2_O_2_, MCF-7 and Caucasian gastric adenocarcinoma cancer cells, Therapeutics efficiency

## Abstract

**Background:**

Given the great benefits of artificial enzymes, a simple approach is proposed via assembling of Ni^2+^ with hemin for synthesis of Ni-hemin metal–organic-frameworks (Ni-hemin MOFs) mimic enzyme. The formation of the Ni-hemin MOFs was verified by scanning electron microscopy, Transmission electron microscopy, X-ray powder diffraction, X-ray photoelectron spectroscopy, Fourier transform infrared spectroscopy, Energy-dispersive X-ray spectroscopy and UV–vis absorption spectroscopy. This novel nanocomposite exhibited surprising peroxidase like activity monitored by catalytic oxidation of a typical peroxidase substrate, 3,3,5,5′-tetramethylbenzidine, in the presence of H_2_O_2_. By using folic acid conjugated MOF nanocomposite as a recognition element, we develop a colorimetric assay for the direct detection of cancer cells.

**Results:**

The proposed sensor presented high sensitivity and selectivity for the detection of human breast cancer cells (MCF-7) and Human Caucasian gastric adenocarcinoma. By measuring UV–vis absorbance response, a wide detection range from 50 to 10^5^ cells/mL with a detection limit as low as 10 cells/mLwas reached for MCF-7 cells. We further discuss therapeutics efficiency of Ni-hemin MOFs in the presence of H_2_O_2_ and ascorbic acid. Peroxidase-mimic Ni-hemin MOFs as reactive oxygen species which could damage MCF-7 cancer cells, however for normal cells (human embryonic kidney HEK 293 cells) killing effect was negligible.

**Conclusions:**

Based on these behaviors, the developed method offers a fast, easy and cheap assay for the interest in future diagnostic and treatment application.

## Background

Lately, artificial enzyme mimics are becoming a focus of great interest owing to the remarkable superiority that they offer over natural enzymes, such as highly stable and low-cost [1–3] nanozymes (nanomaterials with enzyme-like characteristics) generally exhibit superior catalytic activity and intrinsic ability to generate or scavenge reactive oxygen species due to their small size and large surface area [4]. Since Gao et al. [5] reported that inert ferromagnetic nanoparticles with intrinsic horseradish peroxidase (HRP)-like activity, a numerous deal of excellent work about the enzyme mimics has been done, including CeO_2_ nanoparticle [6], Co_3_O_4_ Nanorods [7], MnO_2_ nanoparticles [8], MoS_2_ nanosheet [9], CuO nanoparticles [10], nitrogen-doped graphene quantum dots [11], Hematite-Silica Nanoparticles [12], and metal organic frameworks [13].

Metal–organic frameworks (MOFs) formed by self-assembly of metal ions and organic linkers, have recently emerged as new versatile materials. MOFs have received special interest owing to their attracting features including large specific surface area, large pore volume and high stability [14, 15]. Because of unique characteristics of MOF, they have potential applications as functional materials in catalysis [16], separations [17], electronic [18], drug delivery [19], and sensing [20, 21].

Recently, some studies have been reported on MOF based biosensing. MIL-53(Fe) with intrinsic peroxidase-like catalytic activity can catalyze the oxidation of different peroxidase substrates such as 3,3′,5,5′-tetramethylbenzidine (TMB) and *o*-phenylenediamine (OPD) in the presence of H_2_O_2_ providing a new and simple colorimetric detection of hydrogen peroxide and ascorbic acid [22]. Metalloporphyrinic MOF is also a type of MOF which used as biomimetic catalysts for different reactions. Huang and co-workers grown Au nanoparticles (NPs) on 2D metalloporphyrinic-MOF nanosheets. This synthesized hybrid could act as the glucose oxidase (GOx) mimics and utilized to detect glucose [23].

Hemin, as one of the iron porphyrin derivatives, is the active center of heme-proteins, having abilities to mimic the active site of various enzymes. Due to molecular aggregation of hemine in aqueous solution to form catalytic inactive dimers and oxidative self-destruction in the oxidizing media, direct application of hemin is of significant challenge [24]. Therefore, the development of novel materials such as hemin supports to achieve biomimetic catalysts with enzyme-like activity is highly desired. So, various nanomaterials have been used as hemin support for improving its enzyme like activity. He et al. [25] have been introduced Cu-hemin metal–organic frameworks as a kind of Metalloporphyrinic MOF and applied for electrochemical glucose biosensing. This ball-flower-like nanostructure with excellent catalytic activity toward the reduction of O_2_ was synthesized via Cu^2+^ coordinating with hemin, possess and used to load a large number of glucose oxidase (GOD) molecules. The pores of MOFs provide incorporating of GOD molecules into Cu-hemin MOFs which effectively avoided the aggregation of enzyme on the surface of electrode. In addition, Cu-hemin MOFs can be employ to colorimetric detection of H_2_O_2_ and glucose [26]. Nevertheless, there have been a few studies on usage of the intrinsic enzyme-like activities of MOF for biomedical applications such as cancer diagnostic [27].

Cancer is one of the killer sickness in the world and nowadays it has become a major public worry. Hence, it is highly required to develop rapid, sensitive and specific methods to identification and detection cancers [28–30]. Among various techniques of cancers diagnosis like cytologic testing [31], fluorescent imaging [32], X-ray imaging [33], low-cost and non-destructive methods are preferred for preclinical detection of cancer cells. Therefore, colorimetric methods have attracted significant attention for developing cytosensing platforms because they can reduce the cost and time required for analysis and usually performed with simple instrumentation [34, 35].

In this paper, we developed a facial approach to fabricate a new Ni-hemin MOF nanostructure via a one-step hydrothermal method for sensitive colorimetric detection of cancer cells. To the best of our knowledge, it is the first time that this type of the MOFs are used for the colorimetric detection of cancer cells. The resulting Ni-hemin metal–organic framework exhibited intrinsic peroxidase-like activity and catalyze the oxidation of 3,3′,5,5- tetramethylbenzidine (TMB) by hydrogen peroxide. Therefore, the MOF nanocomposite conjugated with folic acid as cancer cell targeting ligand. The prepared immobilized nanocomposite was obtained to use as a powerful nanoprobe for sensitive and selective colorimetric detection of cancer cells, moreover for therapeutic cancer treatment through great peroxidase activity of the nanocomposite.

## Methods

### Materials and instrumentation

Nickel nitrate hexahydrate Ni(NO_3_)_2_.6H_2_O, cetrimonium bromide (CTAB), phytic acid (C_6_H_18_O_24_P_6_), hemin (C_34_H_32_ClFeN_4_O_4_), were purchased from Sigma or Aldrich. NH_3_, H_2_O_2_ (30%), 3,3ʹ,5,5ʹ-tetramethylbenzidine (TMB), folic acid (FA), (3-Aminopropyl) triethoxysilane (APTES) and all other reagents of analytical grade were from Merck or Fluka. All chemicals and reagents were of analytical grade with the highest purity and directly used without further purification. Deionized water from a Milli-Q Plus system (Millipore) was used in all solutions and experiment. UV–visible absorption spectra and kinetic measurements were carried out on a SPECTROD 250-analytikjena spectrophotometer (Germany). Scanning electron microscopy (SEM) and Transmission electron microscopy (TEM) image were recorded on a MIRA3 TESCAN HV: 20.0 kV from Czech Republic and Philips microscope (EM 280, Tokyo, Japan) respectively. X-ray diffraction (XRD) patterns were observed using a Bruker D8 Advance diffractometer equipped with a copper source and a general area detector diffraction system (GADDS). Netherlands). X-ray Photoelectron Spectroscopy (XPS) was recorded using Thermo Scientific ESCALAB 250 spectrometer with a mono X-ray source Al K a excitation (1486.6 eV). Binding energy calibration was based on C1 s at 284.6 eV.

### Synthesis of Ni-hemin metal–organic framework

Ni-hemin MOFs Nanocomposites were prepared as follows: Briefly, 0.28 g Ni(NO_3_)_2_.6H_2_O was dispersed in 20 mL of deionized water, then 0.055 g CTAB and 0.045 phytic acid was added to solution and stirred at room temperature for 15 min. Subsequently, 20 mL of 0.5 mM hemin was mixed with the above solution, followed by adding 2 mL of ammonia solution. After continuous stirred for 10 min, the product was then transferred to a 40-mL Teflon-lined stainless steel autoclave and kept in an oven at 150 °C for 8 h. The autoclave was then taken out from the oven and left to cool to room temperature. The sediment was collected via centrifugation, washed thoroughly several times and dried at 60 °C overnight.

### Preparation of folic acid/Ni-hemin MOF

Firstly, 100 μL APTES was added into 1 mL prepared Ni-hemin MOF (1 mg/mL) solution and stirred for 8 h at room temperature. After centrifugation to remove unbounded APTES, the sediment was resuspended in 1 mL PBS (pH 7.0). In the next step, 2 mL of folic acid (0.5 mg mL^−1^) was added to a solution containing EDC (2 mL, 1 mg mL^−1^) and NHS (2 mL, 0.5 mg mL^−1^) and stirred for 1 h at room temperature. The solution of folic acid was then mixed with APTES modified Ni-hemin MOF solution and stirred mechanically overnight. The obtained folic acid functionalized Ni-hemin MOF was subjected to centrifugalize at 3000 rpm for 10 min and washed several times by ultrapure water. Finally, the precipitate was dispersed again in 2 mL PBS (pH 7.0) and kept at 4 °C for the following experiments.

### Peroxidase activity analysis

The catalytic reaction was performed at 25 °C using 50 μg/mL Ni-hemin MOF in a reaction volume of 1 mL phosphate buffer solution (PBS, pH = 7) containing H_2_O_2_ (0.85 mM) and TMB (1.24 mM). The absorbance and the time-dependent absorbance changes at 652 nm within 10 min were assayed. The reaction kinetics of Ni-hemin MOF for the catalytic oxidation of TMB were studied by recording the absorption spectra with selected time interval in scanning kinetics mode. The reaction was performed at room temperature and recorded immediately after the aqueous solution containing desired concentrations of H_2_O_2_ and TMB mixed with 50 μg mL^−1^ Ni-hemin MOF in phosphate buffer solution (PBS, pH = 7). Apparent kinetic parameters were calculated using Lineweaver–Burk plots of the double reciprocal of the Michaelis–Menten equation, 1/V = Km/Vm(1/[S] + 1/Km), where V is the initial velocity, Vm represents the maximal reaction velocity, [S] corresponds to the concentration of substrate, and Km is the Michaelis constant [36].

### Cell culture, bioassay and cell viability evaluation

Human breast cancer cells (MCF-7), Human Caucasian gastric adenocarcinoma (AGS) and human embryonic kidney cells (HEK 293 normal cells) were grown in Dulbecco’s Modified Eagle’s Medium (DMEM) cell culture medium containing 10% fetal bovine serum, penicillin (100 U mL^−1^), and streptomycin (100 mg mL^−1^) under a humidified atmosphere with 5% CO_2_ at 37 °C. For the colorimetric detection, cells were plated into 96-well plate at 37 °C for 1 day. After it was washed with PBS (10 mM, pH 7.4), the cells were fixed with 4% paraformaldehyde at room temperature for 10 min. Afterward, formaldehyde was removed and the cells were again washed with PBS and allowed to incubate with 30 µL of prepared FA/Ni-hemin MOF for 2 h. Then each cell well was washed three times with phosphate buffer solution (0.1 M, pH = 7) to remove unattached FA/Ni-hemin MOF. After that, PBS (0.1 M, pH 7, 200 μL) containing TMB (0.85 mM) and H_2_O_2_ (1.24 mM) was added to each well, and incubated for 20 min at room temperature. Finally, the reaction was terminated by H_2_SO_4_ (0.3 M) and quantitatively measuring were performed for color reaction. The cell viability tests were performed by the standard MTT (3-(4,5-dimethylthiazol-2-yl)-2,5-diphenyltetrazolium bromide) assay method [37]. Briefly, the cells were seeded into 96-well plate (1 × 10^4^ cells per well) in DMEM cell culture medium. After 12 h, the medium was replaced with fresh DMEM (100 μL per well), followed by incubation with different concentrations of Ni-hemin MOF for another 12 h. After washing, H_2_O_2_ (60 or 120 μM) or AA (1 or 2 mM) was added for further 2 h of incubation. The cells were washed with PBS and then cultured for another 18 h. After that, the medium was replaced with DMEM (100 μL per well) containing MTT (0.5 mg mL^−1^) followed by the incubation for 4 h. The medium was removed and the violet frozen crystals were dissolved with DMSO (100 μL). Finally, the absorbance intensity at 565 nm was recorded by a micro plate reader.

## Results and discussion

### Characterization of Ni-hemin metal–organic framework

**Fig. 1:**
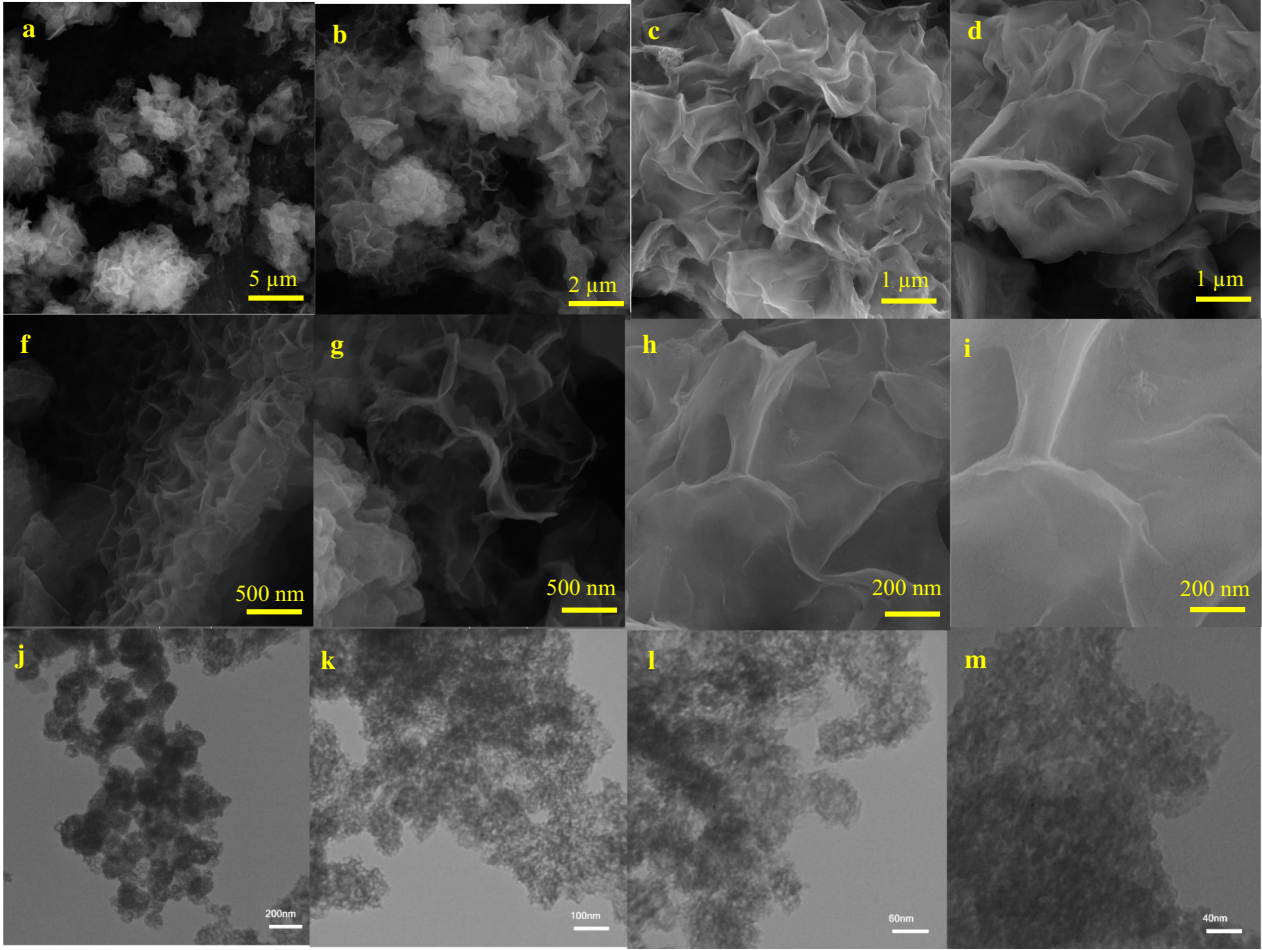
**a**–**i** SEM image and **j**–**m** TEM image of Ni-hemin MOF nanocomposite with different magnitude

**Fig. 2:**
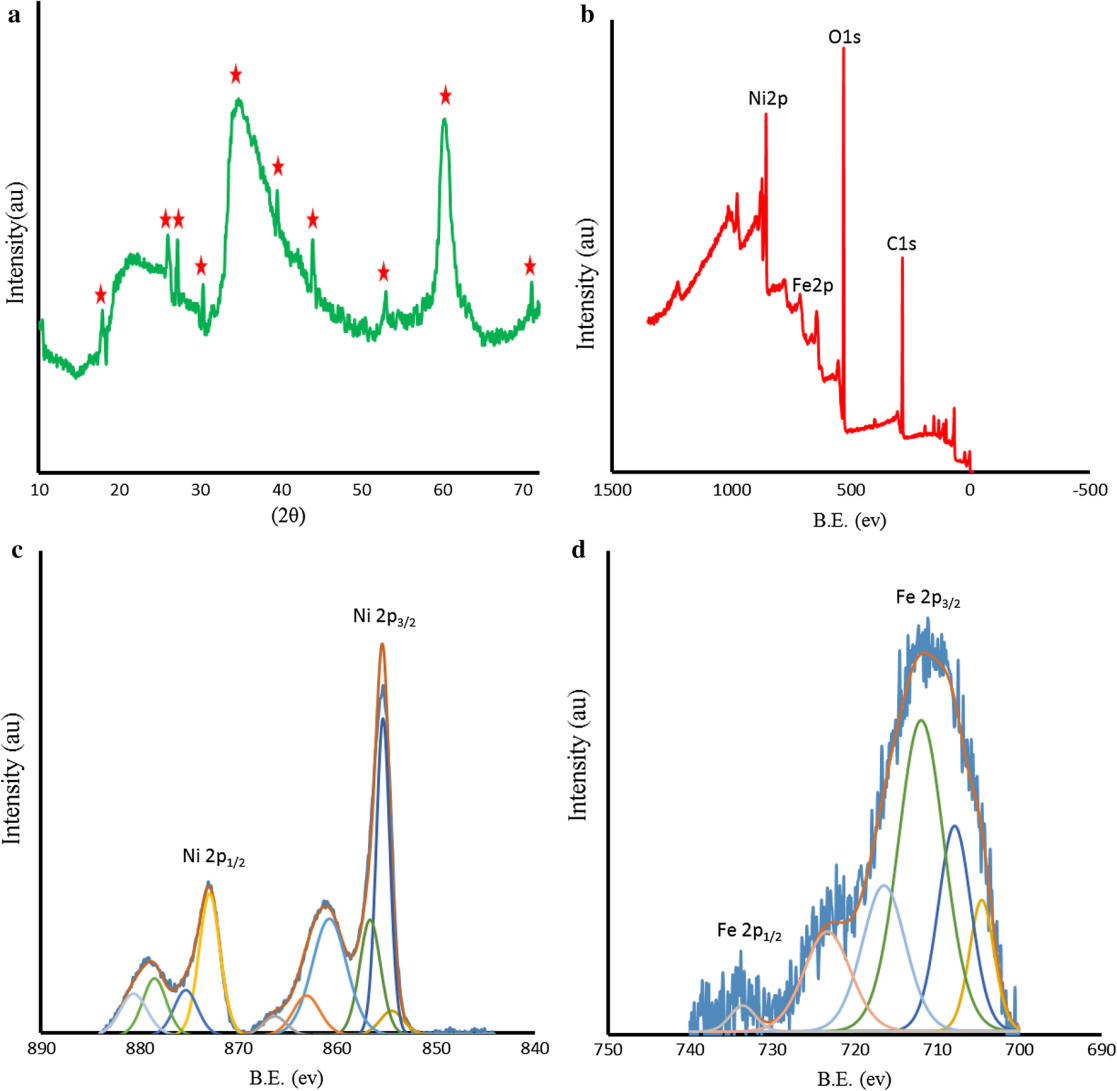
**a** XRD patterns of the Ni-hemin MOF nanocomposite, **b** XPS survey, **c** Ni 2p spectra and **d** Fe 2p spectra of the Ni-hemin MOF nanocomposite

The morphology of the as prepared Ni-hemin MOF nanocomposite was investigated by scanning electron microscopy and transmission electron microscopy (Fig. 1a–i) shows a representative SEM image, from which one can observe a hollow structure of as-prepared MOF composed of wrinkled flakes with an average length of 200 nm–1 µm. Linkages between nickel ions and hemin molecules as an organic ligand provide such morphology which has not yet been reported in previous works on nickel-MOF (Fig. 1j–m) shows a typical TEM image of Ni-hemin MOF, lots of pores on the nanostructure is clearly observed. The characterization of the prepared nanomaterial was also examined with XRD technique. XRD powder pattern of prepared sample are shown in Fig. 2a. The main diffraction peaks of the as-prepared MOF samples appeared at approximately 17.9°, 26.18°, 27.97°, 30.33°, 35.61°, 39.53°, 43.93°, 53.02°,60.30 and 71.78°. The results indicated the as-prepared MOFs material has crystal structure.

**Fig. 3:**
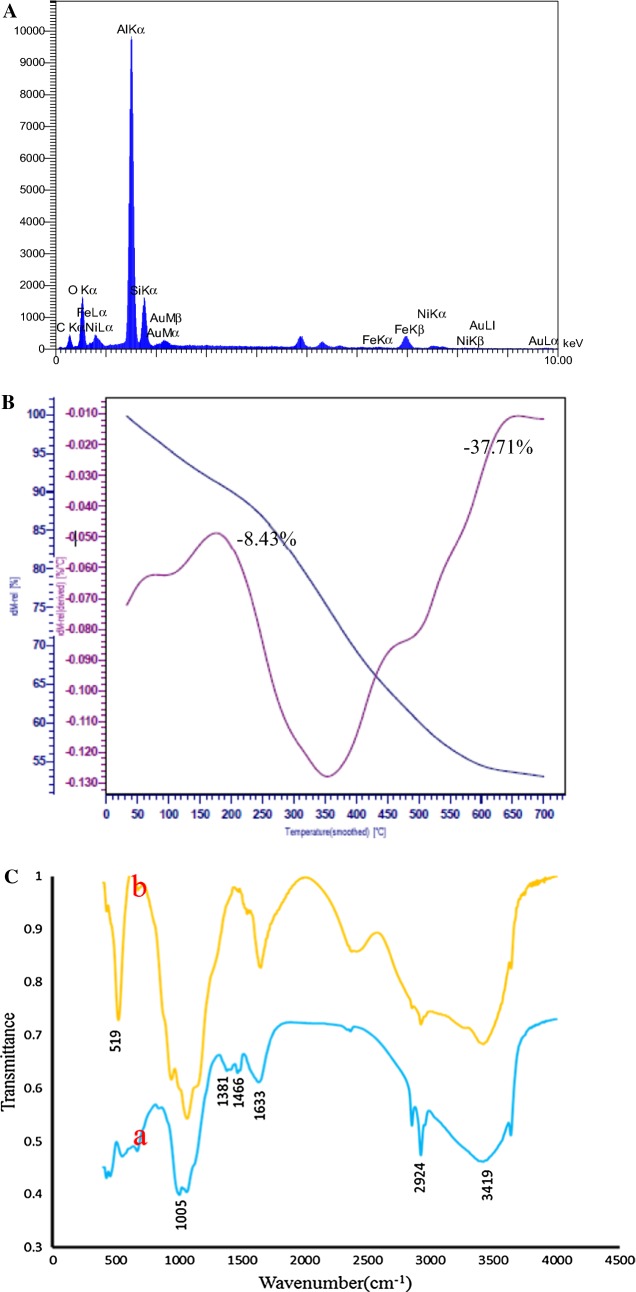
**A** EDS spectra, **B** TGA curve, and **C** FTIR of Ni-hemin MOF nanocomposite

**Fig. 4:**
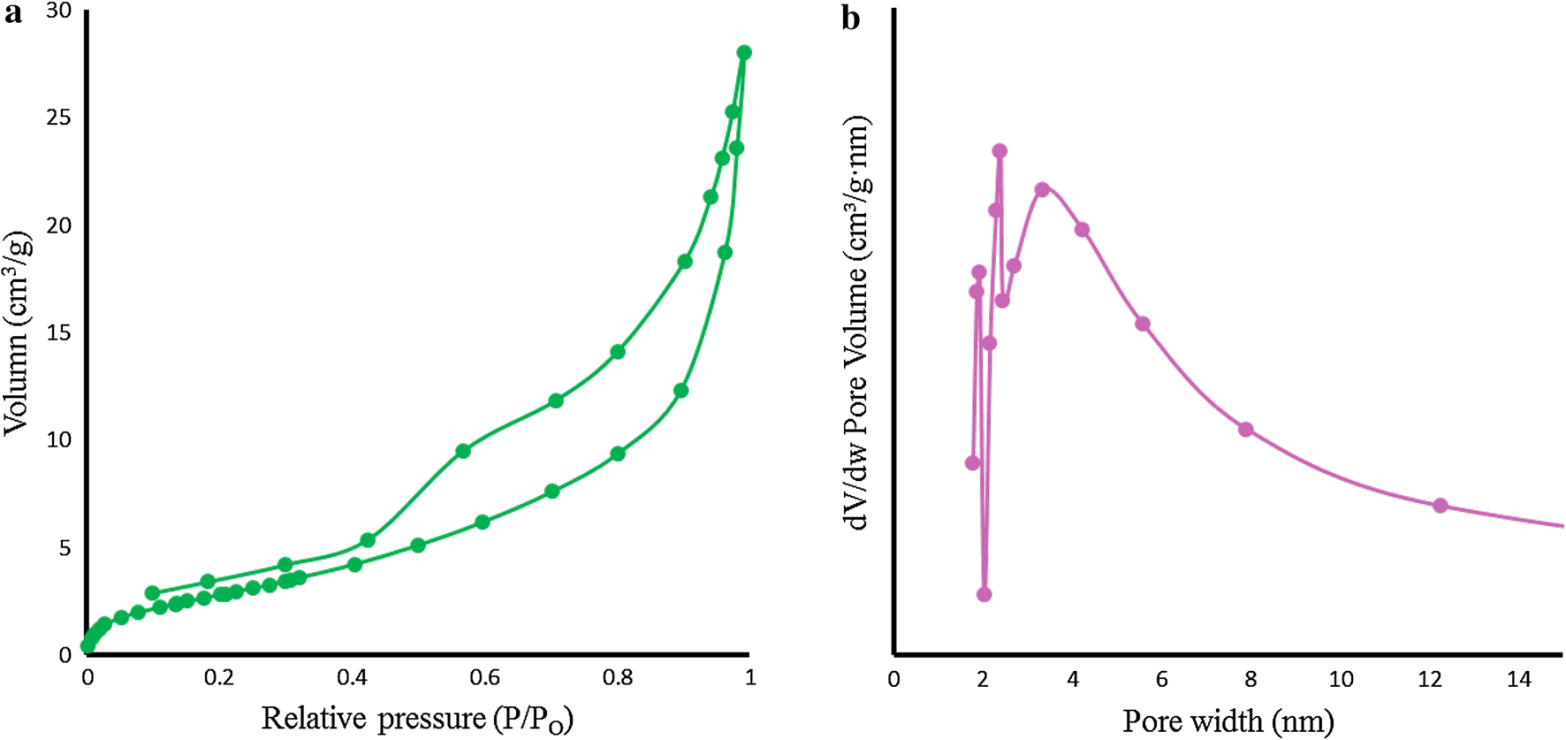
**a** N2 adsorption–desorption isotherm and **b** pore distribution

X-ray photoelectron spectroscopy (XPS) was also accomplished to investigate the chemical states of bonded elements in the as- prepared sample. As can be seen from Fig. 2b, the survey XPS spectra display a set of peaks corresponding with C1 s, O1 s, and Ni2p spectra, respectively. It should be noted that C1 s (285.08 eV) is ascribed to a carbon-based substrate, while the peak at 531.08 eV, 711.98 eV and 855.37 eV are attributed to O1 s, Fe2p and Ni2p spectra, respectively. Figure 2c shows the highly resolved narrow scans Ni 2p spectra of Ni-hemin MOFs, the Ni 2p_3/2_ peak at 855.37 eV are from Ni^2+^ and are associated with the Ni–O octahedral bonding of cubic Ni-hemin [38]. The high-resolution Fe 2p XPS spectrum revealed that curve-fitted into two peaks (Fig. 2d), the peak at 711.98 is relating to Fe^3+^ of hemin in Ni-hemin MOF. The Ni and Fe content in Ni-hemin MOFs was 13.38 and 6.19%, respectively, based on XPS. The EDS analysis of Ni-hemin MOFs also clearly revealed their elemental composition and corroborated the presence of Ni and Fe in prepared nanocomposite (Fig. 3A). TGA analysis further executed and the result shown in Fig. 3B. Typically, TGA curve shows a little weight loss peak at the temperature range of 35–170 °C and an obvious peak at 170–650 °C. The weight losses corresponding to the two steps in TGA are about 8.43 and 37.71%, respectively. The first weight loss ascribes to the removal of structural water, and the second due to the removal of carbon structure of hemin. FT-IR spectra can provide some useful information on the structure of Ni-hemin MOFs nanocomposite. Figure 3C shows the comparative FT-IR spectra of Ni-hemin MOFs and FA/Ni-hemin MOFs nanocomposite. As shown in curve a, the FT-IR spectra of Ni-hemin MOFs shows the peaks at 3419 cm^−1^and 2924 cm^−1^ originated from O–H and C-H of hemin. The absorption band assigned to the C=O stretch mode of carboxylic group at 1633 cm^−1^, an absorption band at 1466, 1381 cm^−1^and 1005 cm^−1^ attributed to the B3u vibration of porphyrin, = C–H deformation vibration of olefin and –CH_3_ of hemin, which confirm the successful formation of porphyrinic-MOF nanocomposite [39]. After immobilization of folic acid on the nanocomposite via APTES (curve b), the characteristic and absorption at 519 cm^−1^ for vibrations from Ni-Si–O has appeared which, attributed to the conjugation of APTES. Furthermore, the absorption band of carboxylic group was stronger in comparison with Ni-hemin MOFs, confirming successful modification of nanocomposite with folic acid. N_2_ adsorption‐desorption isotherms measurements were performed to investigate the porosity of the obtained Ni-hemin MOFs. As can be seen from Fig. 4a the isotherms of Ni-hemin MOFs shows a typical IV isotherm contained a hysteresis loop, which indicates the being of different pore sizes distribution ranged from micro-to mesopores [40]. Figure 2b also shows pore diameter distribution of nanocomposite which is mainly 1.91, 2.38 and 3.31 nm.

### Peroxidase-like activity of Ni-hemin MOFs

**Fig. 5:**
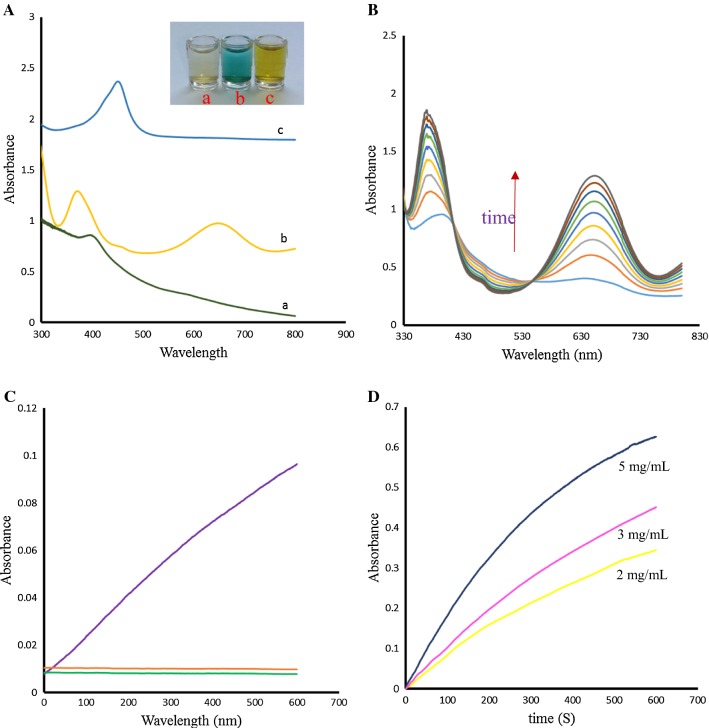
**A** UV–vis and photographs of (a) Ni-hemin MOF solution (b) Ni-hemin MOF +TMB + H_2_O_2_ (c) Ni-hemin MOF +TMB + H_2_O_2_ +H_2_SO_4_, **B** Time-dependent UV–vis spectral changes of TMB solution with H_2_O_2_ catalyzed by NiO MOF, **C** Time-dependent absorbance changes of (a) Ni-hemin MOF solution (b) TMB + H_2_O_2_ (c) Ni-hemin MOF +TMB + H_2_O_2_ at 652 nm, and **D** Time-dependent absorbance changes of TMB solution with H_2_O_2_ at 652 nm in the presence of different concentrations of NiO MOF

To demonstrate that Ni-hemin MOFs is an ideal biomimetic catalyst, we evaluated the peroxidase-like activity of Ni-hemin MOFs by catalytic oxidation of the common peroxidase substrate, TMB, in the presence of H_2_O_2_ (Fig. 5A). As can be observed in UV–vis spectra and photographs, the Ni-hemin MOFs aqueous solutions exhibited almost no color change without H_2_O_2_ and TMB, although an absorption peak at 400 nm indicated the presence of hemin in nanocomposite (curve a). When solution of TMB and H_2_O_2_ was introduced into the Ni-hemin MOFs, a typical blue color appeared in the solution (curve b). The new absorption peaks appeared at 650 nm and 373 nm which is attributed to the charge-transfer complexes derived from the one electron oxidation of TMB (TMB ox) [41] and the intensity of these peaks increased with increasing of reaction time (Fig. 5B).

**Fig. 6:**
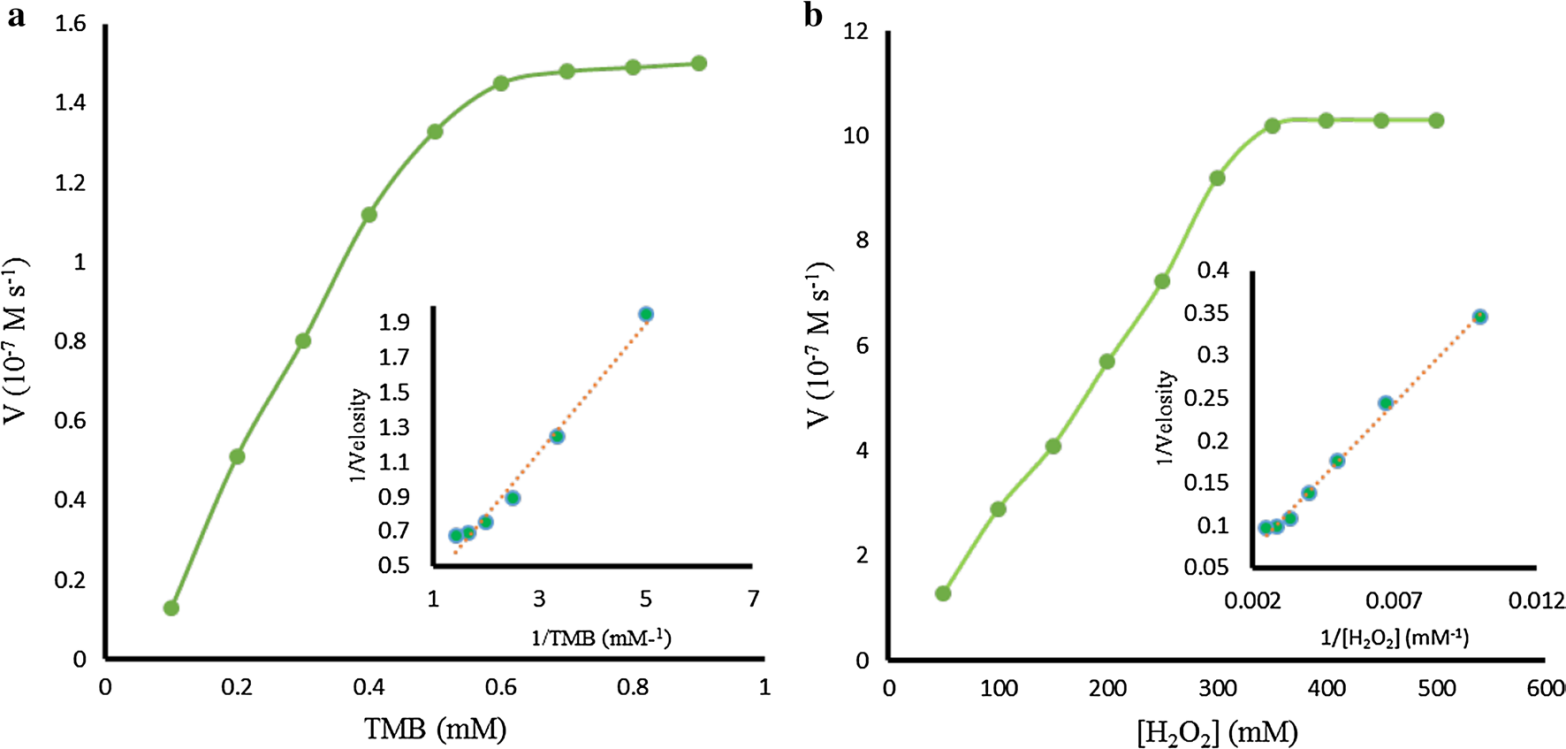
Steady-state kinetic analyses using the Michaelis–Menten model and Lineweaver–Burk model (insets) for Ni-hemin MOF nanocomposite by **a** varying the concentration of TMB with a fixed amount of H_2_O_2_ and **b** varying the concentration of H_2_O_2_ with a fixed amount of TMB

Upon addition of H_2_SO_4_, the color of solution turned to yellow and absorption peak was observed at 450 nm (Fig. 5A, curve c). Comparison experiments with (a) Ni-hemin MOFs (b) TMB and H_2_O_2_ (c) Ni-hemin MOFs + TMB and H_2_O_2_ were performed by measuring the time-dependent absorbance at 652 nm (Fig. 5c), the significant peroxidase catalytic activity was observed for Ni-hemin MOFs. It was found that the catalytic activity increased with increasing the amount of the Ni-hemin MOFs nanocomposite (Fig. 5D). The apparent steady-state kinetic parameters for the peroxidase- like enzymatic color reaction were determined by varying the concentrations of TMB and H_2_O_2_ in the system. Typical Michaelis–Menten curves can be obtained for the Ni-hemin MOFs with both TMB and H_2_O_2_ (Fig. 6a, b). Firstly, time scanning curves was obtained with a UV–Vis spectrophotometer at the wavelength of 652 nm, then, a set of initial velocities calculated and converted with molar absorption coefficient of TMB oxidation products (*ε*652 nm = 3.9 × 104 M^−1^ cm^−1^) [42]. Typical double reciprocal plots, 1/ν vs. 1/[S] (Fig. 6a, b insets), were constructed and fitted to the Michaelis–Menten equation to calculate the catalytic parameters Km and Vm given in Table 1. The Michaelis constants (Km) of the Ni-hemin MOFs for TMB and H_2_O_2_ were determined to be 0.006 mM and 2.31 mM, respectively. In comparison with the natural enzyme HRP the Ni-hemin MOFs have a relatively lower *Km* value for TMB and H_2_O_2_, which indicate (Km for HRP) the higher affinity of the proposed MOF compared to natural HRP. So, peroxidase-like Ni-hemin MOFs shows relatively high affinity for both TMB and H_2_O_2_.

**Table 1:** Kinetic Parameters of Ni-hemin MOF hybrid and HRP Obtained from Their Corresponding Michaelis–Menten Curves

Catalyst	Substance	Km (mM)	Vmax (_˟_10^−7^M.s^−1^)
Ni-hemin MOFs	TMB	0.006	18.51
Ni-hemin MOFs	H_2_O_2_	2.31	36.66
HRP	TMB	0.43	10
HRP	H_2_O_2_	3.70	8.7

### Colorimetric detection of cancer cells

**Fig. 7:**
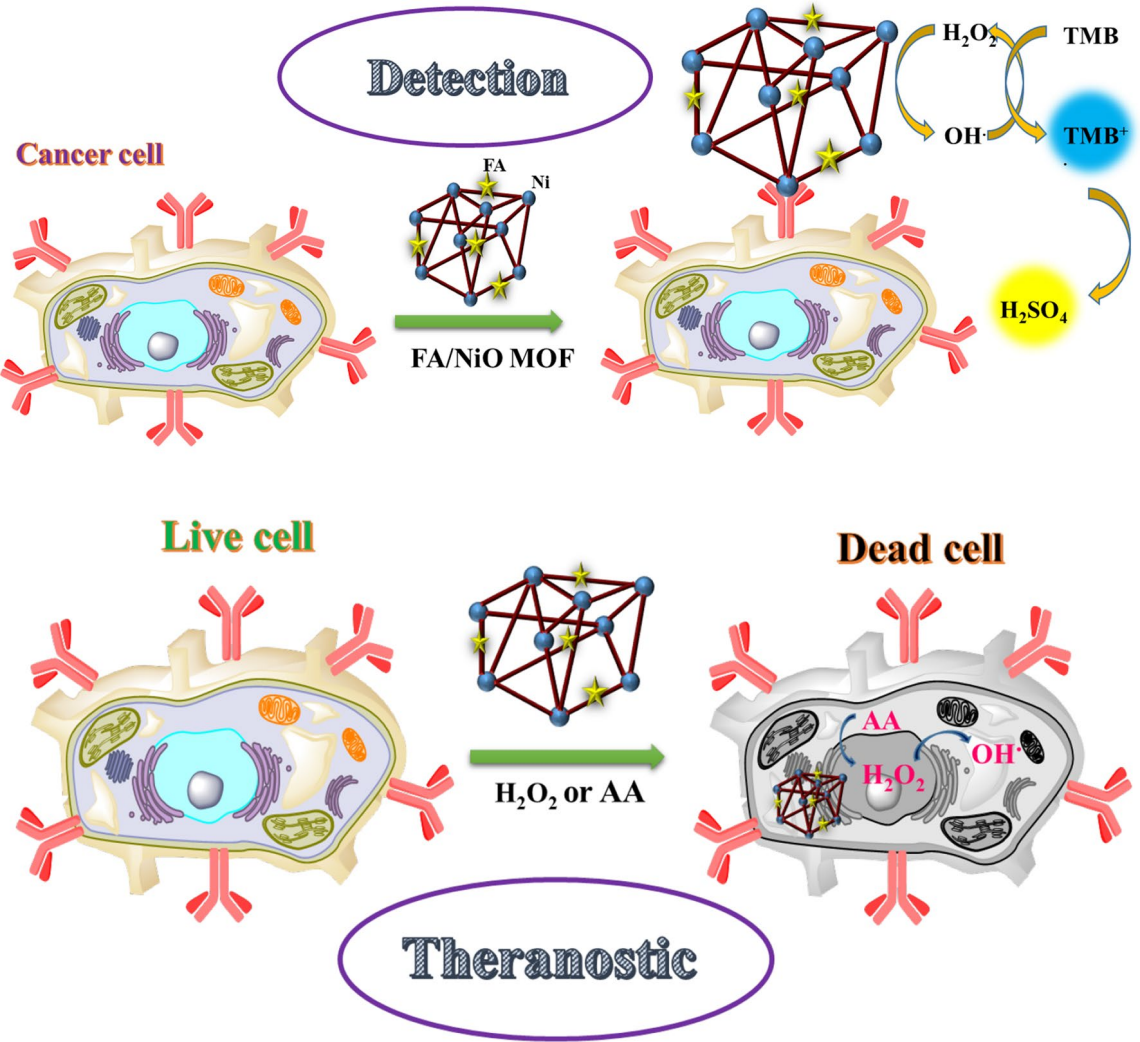
Schematic illustration of peroxidase activity of Ni-hemin MOF for cancer cell detection

**Fig. 8:**
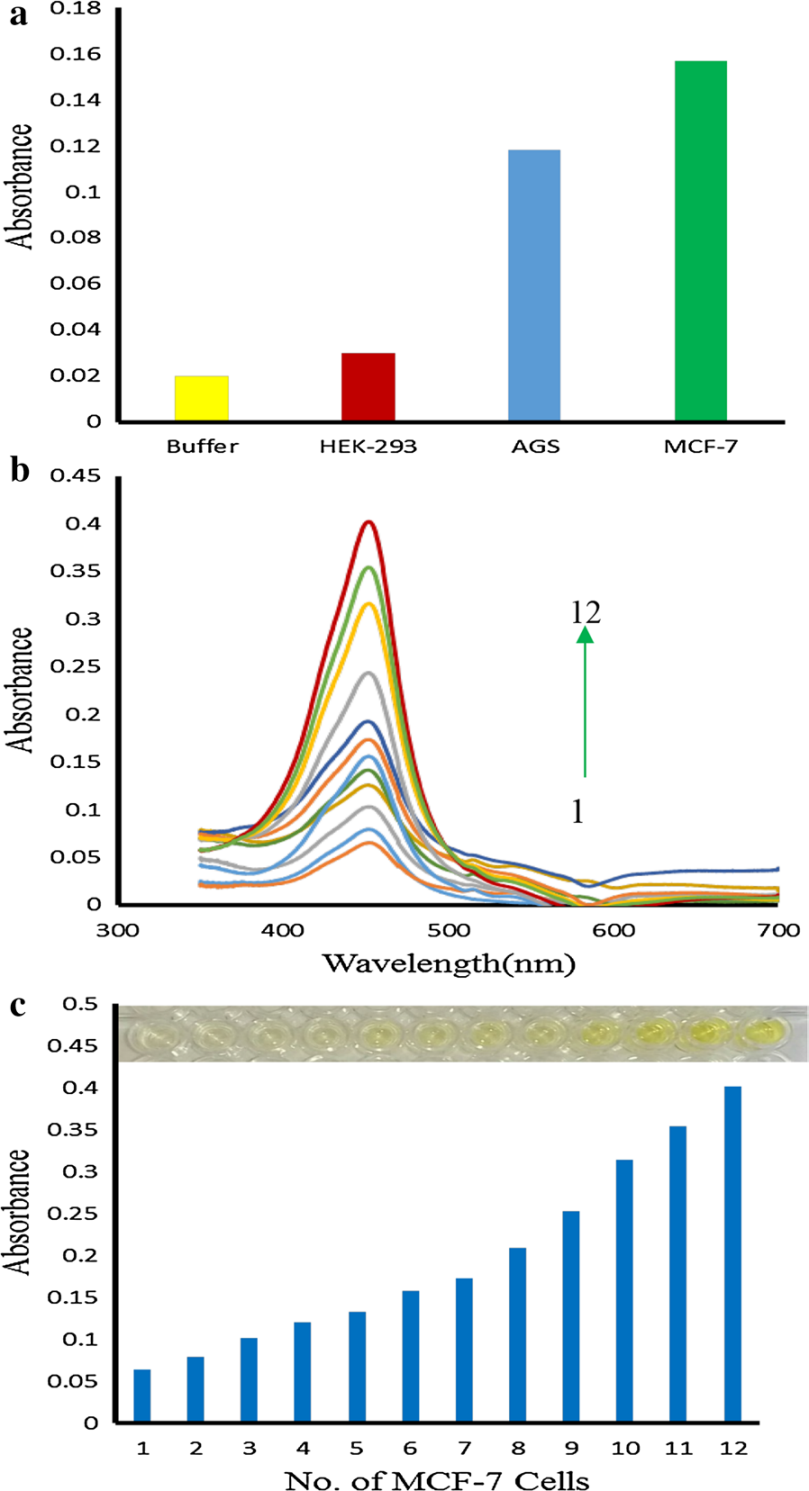
**a** Target-directed cancer cell detection **b** Uu-vis absorbance changes at 450 nm upon analyzing different numbers of MCF-7 cells (1) 5 × 10^1^, (2) 1 × 10^2^, (3) 2 × 10^2^, (4) 4 × 10^2^, (5) 8 × 10^2^, (6) 1.6 × 10^3^, (7) 3.2 × 10^3^ (8) 6.4 × 10^3^, (9) 1.3 × 10^4^, (10) 2.5 × 10^4^, (11) 5 × 10^4^, and (12) 1 × 10^5^, **c** the absorption intensity changes at 450 nm and photographs of the well plates (inset) with corresponding different numbers (50−1 × 10^5^) of MCF-7

In our investigations, FA/Ni-hemin MOF with superior peroxidase activity was used as the signal indicator for cancer cell detection. As aforesaid, selective targeting of hybrid material with cancerous cells occurs through attached FA targeting ligand [43]. TMB as substrate agent molecule lost one electron and transformed into the status of cation radical, then the colorless solution turned to blue color. Then the oxidation reaction stopped by H_2_SO_4_ leading to the cation radical of TMB molecule, which further lost another electron to form diamine, and the blue solution changed to be yellow [44]. This working principle is demonstrated in Fig. 7. Herein, we chose human breast cancer cells (MCF-7) and Caucasian gastric adenocarcinoma (AGS) expressing folate receptors on the cell membrane, and a normal cell line human embryonic kidney cells (HEK 293) with lack of folate receptors as a control [45]. The four samples are as follow: 30 µL of FA/Ni-hemin MOF with no cells, 30 µL of FA/Ni-hemin MOF with 3200 HEK 293, 30 µL of FA/Ni-hemin MOF with 3200 AGS cells, 30 µL of FA/Ni-hemin MOF with 3200 MCF-7 cells. After the addition of TMB-H_2_O_2_ and subsequently H_2_SO_4_ solution, the specific generation of yellow color was observed in the case of MCF-7 and AGS cells, whereas for the wells containing HEK 293 cell and buffer medium without any cell lines, no significant color change was observed (Fig. 8a). These results indicate that the FA functionalized Ni-hemin MOF (FA/Ni-hemin MOF) is a good recognition element and could differentiate between target cells and control cells. Moreover, because of the different amounts of folate receptor expression on different types of cancer cells, more FA/Ni-hemin MOF nanocomposites are bound to MCF-7 cells more than AGS cells. To further evaluate the capability of the hybrid for calorimetrically differentiate between different numbers of cancer cells, a suitable amount of FA/Ni-hemin MOF was incubated with various concentrations of MCF-7 cells in a 96 well plate. Figure 8b, c shows the image of color variation and UV–vis absorbance changes at 450 nm and the image of color variation in the presence of varying amounts of MCF-7 cells. Color variation of TMB oxidation and the absorbance change at 450 nm is proportional to the number of MCF-7 cells over a range of 50–10^5^ cells. This color change of the solution was obvious and easily observed by the naked-eye. The assay has appropriate sensitivity to cancer cells, the limit of detection of MCF-7 cells was calculated to be 10 cells, which is lower than that of previously reported cancer cell detection [46–48]. Thus, the proposed diagnostic system based on FA/Ni-hemin MOF probe is a rapid, cost-effective, sensitive and selective method for the accurate and early detection of cancer cells.

### Cell viability assay (MTT assay)

**Fig. 9:**
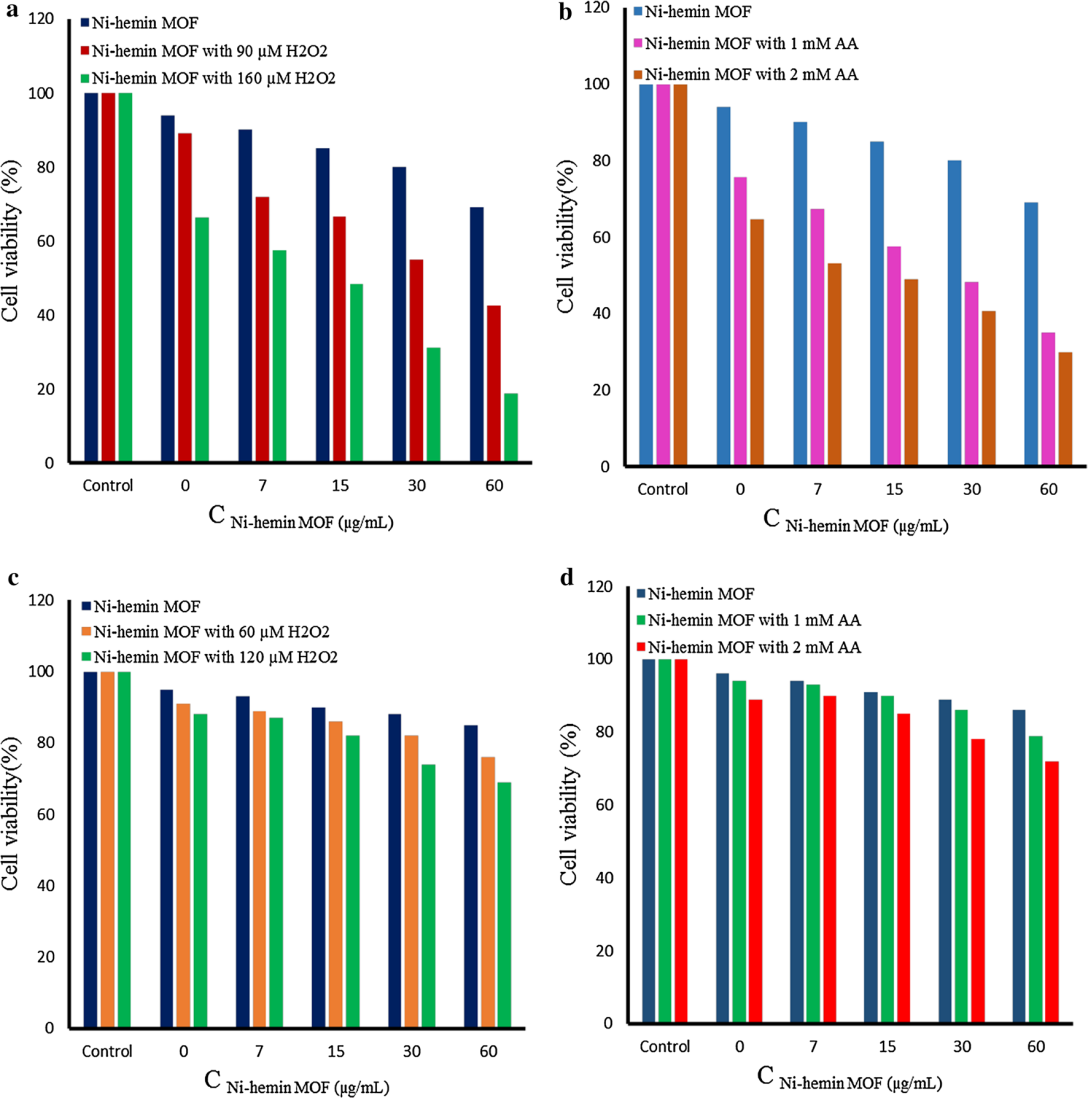
**a** Cell viability tests by MTT assay for MCF-7 cells in the presence of (**a**) Ni-hemin MOF with H_2_O_2_ (90 or 160 μM) and **b** Ni-hemin MOF with AA (1 or 2 mM). **c** HEK-293 cell viability upon treatment by Ni-hemin MOF with H_2_O_2_ (90 or 160 μM) and **d** Ni-hemin MOF with AA (1 or 2 mM)

Previous studies shown that the nanozymes with peroxidase-like activity could generate strong oxidant.OH via decomposition of H_2_O_2_ [49]. According to this phenomenon, we utilized Ni-hemin MOF nanocomposites for therapeutic treatment of cancer cells based on its efficient generation of OH• radical through the peroxidase activity. Therefore, to evaluate anticancer activity, we incubated various concentrations of Ni-hemin MOF (7–60 μg mL^−1^) with MCF-7 cells for 12 h. Firstly, inherent toxicity of nanocomposite was measured using MTT (3-(4,5-dimethylthiazol-2-yl)-2,5-diphenyltetrazolium bromide) assay, showing a slight cytotoxicity [50]. Then, H_2_O_2_ (90 or 160 μM) was added into the cell lines, and the cell viability was investigated. As shown in Fig. 9a, with increasing concentration of both Ni-hemin MOF and H_2_O_2_ the cell viability significantly decreased up to ∼ 18%. indicating the promising anticancer capability of the prepared nanocomposite. Ascorbic acid (AA) as an antioxidant could produces endogenous H_2_O_2_ to result in generation of oxidant.OH. So, in this research, instead of using exogenous H_2_O_2_, 1 or 2 mM AA was applied along with Ni-hemin MOF (7–60 μg mL^−1^) to evaluate the cancer treatment strategy. As displayed in Fig. 9b, the cell viability decreased remarkably to ∼ 29% in the presence of Ni-hemin MOF and AA in a concentration dependent manner. To check the targeted therapeutic ability of the nanocomposite to effect only the cancer cells, normal HEK 293 cells were also treated under same conditions and the cell viability was determined. Compared to cancer cells a minor cytotoxicity was determined for Ni-hemin MOF along with H_2_O_2_ (∼ 31% cell death at high concentration) and AA (∼ 28% cell death at high concentration), respectively (Figure 9c, d). This result implicating the selective targeting ability of the nanocomposite to MCF-7 cancer cells through the FA targeting ligand.

## Conclusions

In this work, we have obtained the new kind of metal organic framework nanostructure (Ni-hemin MOF) via simple process and demonstrated that Ni-hemin MOF nanocomposites possess high intrinsic peroxidase-like activity. Peroxidase-like activity of Ni-hemin MOFs was investigated by catalytic oxidation of the common peroxidase substrate, TMB, in the presence of H_2_O_2_ (Fig. 4). Ni-hemin MOFs could oxidize TMB in the presence of H_2_O_2_ and produce color solution. Apparent kinetic parameters were obtained using Lineweaver–Burk plots of the double reciprocal of the Michaelis–Menten equation, 1/V = Km/Vm(1/[S] + 1/Km) (Fig. 5). Ni-hemin have a relatively lower *Km* value for both substrate TMB and H_2_O_2_. As compared to natural enzymes, Ni-hemin MOF have several advantages such as low-cost and high-stability and very high substrate-binding affinity as compared to HRP. On the basis of folic acid ability to recognize elements, the excellent peroxidase activity of Ni-hemin MOF has been utilized for the colorimetric detection of cancer cells. The selective binding Ni-hemin MOF cause significant color change by the oxidation of TMB in the presence H_2_O_2_ for MCF-7 cancer cell detection with a detection limit of 10 cells, which could be also distinguished by the naked eye. Thereby, the prepared FA/Ni-hemin MOF enabled quick colorimetric analysis to give a quantitative and accurate results (Fig. 8). Attractive properties of the novel MOF nanocomposite would be significant to propel the development of novel peroxidase mimics materials. Moreover, this study will facilitate utilization of other recognition elements such as aptamers, antibody, peptide, and nucleic acid to design and develop colorimetric assays in clinical diagnostics and biotechnology. Because of advances in early detection and treatment, the number of cancer survivors continues to grow in the world. Ni-hemin MOF in addition to diagnostic ability, represents a therapeutic activity and significant cancer cell damage was observed through the enhanced generation of OH• radical from exogenous and endogenous H_2_O_2_ mediated by Ni-hemin MOF (Fig. 9). OH• radical as a reactive oxygen species (ROS) causes the damage of cancer cells, however the normal cells have the capability to endure a certain level of exogenous ROS stress. High selectivity of both the detection and therapeutic processes to cancer cell signifying robustness and efficiency of the method.

## Abbreviations

MOF: metal-organic framework
CTAB: cetrimonium bromide
TMB: 3,3ʹ,5,5ʹ-tetramethylbenzidine
FA: folic acid
APTES: (3-Aminopropyl) triethoxysilane
SEM: scanning electron microscopy
TEM: transmission electron microscopy
XRD: X-ray diffraction
XPS: X-ray Photoelectron Spectroscopy
MCF-7: human breast cancer cells
HEK: human embryonic kidney cells
AGS: Human Caucasian gastric adenocarcinoma
